# Secretome Analysis of Mouse Dendritic Cells Interacting with a Probiotic Strain of *Lactobacillus gasseri*

**DOI:** 10.3390/nu12020555

**Published:** 2020-02-20

**Authors:** Maria Fiorella Mazzeo, Diomira Luongo, Toshihiro Sashihara, Mauro Rossi, Rosa Anna Siciliano

**Affiliations:** 1 National Research Council, Institute of Food Sciences, CNR-ISA, Via Roma 64, 83100 Avellino, Italy; fmazzeo@isa.cnr.it (M.F.M.); diomira.luongo@isa.cnr.it (D.L.); mrossi@isa.cnr.it (M.R.); 2 Food Microbiology Research Laboratories, R&D Division, Meiji Co., Ltd., Hachiouji, Tokyo 192-0919, Japan; toshihiro.sashihara@meiji.com

**Keywords:** dendritic cells, immunomodulation, probiotics, proteomics, secreted proteins, mass spectrometry, *Lactobacillus gasseri*

## Abstract

Probiotics play a key role in the modulation of the gut immune system in health and disease and their action is mediated by molecules exposed on the microorganism surface or secreted probiotic-derived factors. In particular, *Lactobacillus gasseri* OLL2809, a probiotic microorganism isolated from human feces, has the potential to modulate various immune responses. The dendritic cells (DCs) are considered the main players in orchestrating the immune response, and their contact with intestinal microbiota is crucial for the development and homeostasis of gut immunity. To gain a perspective on the molecular mechanisms involved in the maturation process of DCs and investigate factors that could modulate these processes, a differential proteomic analysis was performed on the secretome of immature DCs, mature DCs (mDCs, induced by lipopolysaccharide (LPS)), and immature DCs challenged with *L. gasseri* OLL2809 before treatment with LPS (LGmDCs). The maturation process of DCs was associated to profound changes in the protein secretome and probiotic pre-treatment led to a dramatic modulation of several secreted proteins of mDC, not only classical immune mediators (i.e., cytokines, complement factors, T cell Receptor ligands) but also proteins involved in the contractile and desmosome machineries. The latter data highlight a novel mechanism by which *L. gasseri* can modulate the maturation process of DCs, reinforcing the concept of a protective anti-inflammatory role ascribed to this probiotic strain.

## 1. Introduction

Probiotics play a key role in the modulation of the gut immune system in health and disease and their action is mediated by molecules exposed on the microorganism surface or secreted probiotic-derived factors [1]. *Lactobacillus gasseri* OLL2809, a probiotic microorganism isolated from human feces, has the potential to modulate various immune responses [2]. In particular, it is able to enhance the oral tolerance induction and to act as an immune-modulator for the prevention of or early remission from food allergy [3]. The beneficial activity of this strain on mucosal inflammation has also been shown in mice, where the administration of OLL2809 was effective in reducing endometriotic lesions [4]. The immune properties of this microorganism were also exhibited by heat-killed cells that stimulated a high IL-12 (p70) production and a reduction in antigen-specific IgE levels in animal models of allergy [5]. In addition, heat-killed OLL2809 cells had suppressive effects on inflammatory conditions through the inhibition of CD4^+^ T-cell proliferation [6]. We previously found that γ-irradiated *L. gasseri* OLL2809 possessed the ability to modulate the cytokine profile in murine bone-marrow dendritic cells (DCs) [7]. DCs are considered to be major players in orchestrating the immune response, and their contact with intestinal microbiota is crucial for the development and homeostasis of gut immunity. However, the precise mechanisms by which intestinal microbiota can influence the development and function of DCs remain to be further elucidated.

Proteomics have been widely applied to elucidate differences in whole-cell protein patterns of immature DCs (iDCs) and mature DCs (mDCs), and to study exosome proteome, and, recently, phagosomal proteome [8,9,10,11,12,13]. However, only a few proteomic studies have characterized the protein pattern secreted (secretome) by iDCs and mDCs [14,15,16]. Therefore, the secretome analysis could represent an interesting area to exploit to gain a perspective on the molecular mechanisms involved in the maturation process of DCs and investigate factors that could modulate these processes.

Herein, a quantitative proteomic study was performed on the secretome of iDCs, lipopolysaccharide (LPS)-induced mDCs, and mDCs that were obtained from iDCs challenged with *L. gasseri* OLL2809 before inducing the maturation process with LPS (LGmDCs). A label-free quantitative approach based on spectral counting (normalized spectral abundance factor, NSAF) [17] was applied to gain insights into the immunomodulatory properties of *L. gasseri* OLL2809 and thoroughly investigate the cross-talk between dendritic cells and this probiotic microorganism. 

## 2. Materials and Methods

### 2.1. Preparation of L. gasseri

The probiotic strain *Lactobacillus gasseri* OLL28099 was isolated from the human intestine (Patent Microorganisms Depositary, National Institute of Technology and Evaluation, Japan, Accession n. NITE BP-72) [5]. Working cultures were grown in deMan Rogosa Sharpe (MRS) broth (Difco, Detroit, MI, USA) for 24 h at 37 °C under microaerobic conditions. For co-culture experiments, bacterial strains were harvested in the late log phase and irradiated with 2800 Gy (Gray) γ-irradiation (MDS Nordion γ-cell 1000, Nordion, Ottawa, ON, Canada). This treatment completely inactivated the *L. gasseri* preparations.

### 2.2. Dendritic Cell Preparation and Co-Culture

BALB/c mice were maintained in pathogen-free conditions at our animal facility (accreditation no. DM.161/99). Animal studies were approved by the review committee of Health Ministry, General Division of Animal Health and of Veterinary Medicine (2016) and performed according to European regulations (EU Directive 2010/63/EU). Murine DCs were generated according to a previously published method [18]. In brief, bone marrow cells were collected in 10 mL RPMI 1640 medium (Sigma, St. Louis, MO, USA) containing antibiotics (penicillin 100 IU/mL; streptomycin 100 IU/mL, Sigma), 10% fetal calf serum (Sigma), 1% non-essential amino acids (EuroClone, Milan, Italy), and 20 ng/mL granulocyte–macrophage colony-stimulating factor (GM-CSF; PharMingen, San Diego, CA, USA) (complete medium). On day 3, 10 mL complete medium was added; on day 7, the 10 mL medium was replaced with fresh medium. This procedure eliminated all other bone marrow cells.

To obtain a comprehensive picture of the protein secretome from murine bone marrow DCs, protein contribution from cell culture medium was drastically reduced by incubating cells under serum-free conditions [18]. Specifically, on day 9, non-adherent immature DCs (iDCs) were harvested, placed in 24-well plates (1 × 10^6^ cells/mL) and transferred in X-Vivo 15 medium (Lonza, Basel, Switzerland). iDCs were then incubated for 24 h with irradiated bacteria re-suspended in X-Vivo 15 medium at a 30:1 (bacteria: eukaryotic cell ratio). Finally, 1 μg/mL lipopolysaccharide (LPS) was added to the culture for 6 h to induce the maturation of DCs. Following incubation, cell viability was microscopically evaluated by a dye-exclusion test using Nigrosin (1% solution) and found ≥ 90% live cells in all experiments. To estimate the residual percentage of lysed cells in culture supernatants, Lactate Dehydrogenase (LDH) assay was used [19]. Results were expressed as a percentage of total LDH release from control cultures treated with 1% (*w*/*v*) NP-40 and calculated as follows:

[(experimental value − blank value)/(total lysis − blank value) × 100](1)

### 2.3. Analysis of Cytokine Production

Supernatants from DCs cultures were analyzed for IL-12 and IL-10 protein levels using an in-house sandwich ELISA. One hundred microliters of capture antibody solution (BioLegend, San Diego, CA, USA) were plated into each well of a 96-well plate (NuncMaxisorb, eBioscience Inc., San Diego, CA, USA) and incubated overnight at 4 °C. After antibody removal, 100 μL of PBS supplemented with 1% BSA (blocking buffer) were added to each well and incubated for 2 h at room temperature (RT). Wells were then washed with 0.05% Tween-20 in PBS. Next, cytokine standards (BioLegend, San Diego, CA, USA) or diluted samples were added to the wells and incubated for 2 h at RT. After the washing steps, 100 μL aliquots of biotinylated antibody solution were added and incubated for 2 h at RT. Streptavidin–horseradish peroxidase conjugate solution (1:2000 dilution; BioLegend) was then added to the wells and incubated for 1 h at RT. Finally, after washing, reaction was developed by adding 100 μL of 63 mM Na_2_HPO_4_, 29 mM citric acid (pH 6.0) containing 0.66 mg/mL o-phenylenediamine/HCl and 0.05% hydrogen peroxide. The cytokine concentration was calculated by reading absorbance at 415 nm. Values were expressed as pg/mL.

### 2.4. Fluorescence-activated Cell Sorting (FACS) Analysis

DCs were stained with phycoerythrin (PE)- or fluorescein isothiocyanate (FITC)-conjugated Abs (BioLegend) toward mouse CD80 and CD86 surface markers. Cell staining was analyzed using a CyFlow Space flow cytometer (Partec, Munster, Germany) and FlowJo software (Tree Star Inc., Ashland, OR, USA). For each Ab, an isotype control of the appropriate subclass was used.

### 2.5. Preparation of Samples for Proteomic Analyses

Secreted proteins were extracted from supernatants of iDCs, mDCs, and LGmDCs samples by using a trichloroacetic acid (TCA) precipitation protocol. Four biological replicates were prepared for each sample. TCA and sodium deoxycholate were added to supernatants to 7.5% and 0.1% w/v final concentrations, respectively, and precipitation was carried out for 2 h at 4 °C. Protein pellets, collected by centrifugation (10,000 × *g* for 10 min at 4 °C), were washed twice with 0.5 mL of ice-cold acetone, recovered by centrifugation (10,000× *g* for 10 min at 4 °C) and dried under nitrogen. Pellets were solubilized in 20 mM Tris-HCL, 65 mM DTT, 8 M urea, 2% CHAPS, pH 8.5, and protein concentration was measured by a Bradford assay (BioRad, Hercules, CA, USA). One hundred and twenty microgram aliquots of each sample were loaded on 12% Mini-PROTEAN^®^ TGX™ Precast Protein Gels (BioRad). Gels were run at 120 V using the Mini-PROTEAN Tetra Cell (BioRad). Protein profiles were visualized by staining with Coomassie Brilliant Blue R-250. Each lane was cut in 10 slices that were destained with 50% acetonitrile in 50 mM ammonium bicarbonate. Proteins contained in each slice were submitted to in-gel reduction with 10 mM dithiotreitol in 50 mM ammonium bicarbonate pH 8.0 for 1 h at 56 °C, alkylation with 50 mM iodacetamide in the same buffer for 30 min at RT in the dark and digestion with 0.2 µg modified trypsin (Promega, Madison, WI, USA) in 25 mM ammonium bicarbonate pH 8.4 overnight at 37 °C. Peptide extraction was carried out with 5% formic acid and 50% acetonitrile. Peptides mixtures were dried in a Speed-Vac centrifuge (Savant) and solubilized in 30 µL 0.1% formic acid.

### 2.6. LC-MS/MS Analysis, Protein Identification, and Functional Classification

Tryptic peptide mixtures were analyzed using a Q-Exactive^TM^ mass spectrometer (Thermo Fisher Scientific, Waltham, MA, USA) interfaced with an UltiMate 3000 RSLCnano LC system (Thermo Fisher Scientific). Peptide mixtures were concentrated and desalted on a trapping pre-column (Acclaim PepMap C18, 300 μm × 5 mm nanoViper, 5 μm, 100 Å, Thermo Fisher Scientific), using 0.05% formic acid and 2% acetonitrile at a flow rate of 10 μL/min. The peptide separation was performed at 35 °C using a C18 column (Acclaim Easy Spray PepMap RSLC C18, 75 μm × 15 cm nanoViper, 3 μm, 100 Å, Thermo Fisher Scientific), using as eluent A 0.1% formic acid and as eluent B 80% acetonitrile in 0.08% formic acid at a flow rate of 0.3 μL/min and a linear gradient from 4% to 40% B over 45 min, hold for 10 min, from 40% to 90% B over 1 min, hold for 10 min before column re-equilibration to 4% B.

Mass spectra were acquired in the m/z range 350–1600. Data acquisition was performed in a data-dependent mode Full MS/ddMS2, enabling the acquisition of MS/MS spectra for the ten most intense precursor ions (top ten) and dynamic exclusion of 10 s. The resolution was set to 70,000 for MS spectra acquisition and 17,500 for MS/MS spectra acquisition.

MS data processing for protein identification was performed using ProteomeDiscoverer™ platform (version 2.1.0.81; Thermo Fisher Scientific), interfaced with the Sequest HT Search Engine server (Washburn et al., 2001). The parameters used for the database searches were as follows: Mus musculus protein database (taxon 10,090, downloaded from UniProtKB on June 2018, https://www.uniprot.org/) and a contaminant protein database (PD_Contaminants_2015_5.fasta, provided by the manufacturer), trypsin as proteolytic enzyme, up to two missed cleavages, carbamidomethyl as fixed modification for cysteine residues, oxidation of methionine residues, and formation of pyro-glutamic acid of N-terminal glutamine residues as dynamic modifications, 20 ppm mass tolerance for precursor ions and 0.02 Da mass tolerance for MS/MS fragments. Protein identification based on at least two peptides (FDR < 0.05) was considered reliable. Proteins identified in three out of four biological replicates were considered reproducibly present in one sample.

Quantitative proteomic analysis was carried out using the spectral counting approach to compare protein abundance levels in the secretomes of mDCs vs. iDCs, and LGmDCs vs. mDCs. To calculate each protein normalized spectral abundance factor (NSAF), the number of spectral counts (SpCs; i.e., peptide spectra matches, PSM) was divided by the protein length (thus obtaining the SAF value) and normalized to the sum of SAFs in each sample [17].

*t*-test values were calculated from Log_2_(NSAF) values of the four biological replicates of the compared samples (mDCs vs. iDCs and LGmDCs vs. mDCs). A *p*-value ≤ 0.05 indicated statistically significant differences in protein abundance. Changes in protein abundance (fold change, FC) were calculated as the ratio between the average NSAF of each protein in the two samples. Proteins showing fold changes ≥ ±2 were assumed to be present in different amounts in one of the compared samples. However, fold changes ≥ ±1.5 and *p*-values ≤ 0.1 in these proteins were assumed to give biologically relevant information (Table S1 (quantitation analysis) and Table S2 in Supplementary Materials).

Identified proteins were analyzed using the SignalP 5.0 server (http://www.cbs.dtu.dk/services/SignalP) that predicts the presence and location of signal peptide cleavage sites in amino acid sequences for translocation across cell membranes [20]. Cellular localization of proteins carrying a signal peptide was postulated based on sequence information using DeepLoc 1.0 server (http://www.cbs.dtu.dk/services/DeepLoc-1.0/index.php) [21] and on Uniprot annotation for subcellular localization reported in the UniProtKb database (https://www.uniprot.org). The prediction of non-classically secreted proteins (such as moonlighting proteins) was achieved by the SecretomeP 2.0 server (http://www.cbs.dtu.dk/services/SecretomeP) [22].

Identified proteins were functionally classified using the Reactome Pathway Database (https://reactome.org) that statistically determines whether certain pathways are enriched in the data. This analysis produces a probability score, which is corrected for false discovery rate (FDR) using the Benjamani–Hochberg method [23].

Protein–protein interactions were explored using the web resource STRING v.9.1 (Search Tool for the Retrieval of Interacting Genes/Proteins, http://string-db.org/). Active interaction sources used in our analysis were a neighborhood, co-expression, experiments, gene fusions, gene co-occurrence, databases, and text mining, using high confidence value (0.700) [24].

Gene names for some of the proteins included in functional analyses were deduced using BLASTP (https://blast.ncbi.nlm.nih.gov) (Table S1 (Blast Results) in Supplementary Materials).

### 2.7. Statistical Analysis

Differences among the various treatment groups were determined by the ANOVA test followed by Tukey’s multiple comparisons test. A cut-off of *p* < 0.05 was selected to denote a significant difference.

## 3. Results and Discussion

### 3.1. Phenotypic Characterization and Secretome Analysis of Dendritic Cells

**Figure 1:**
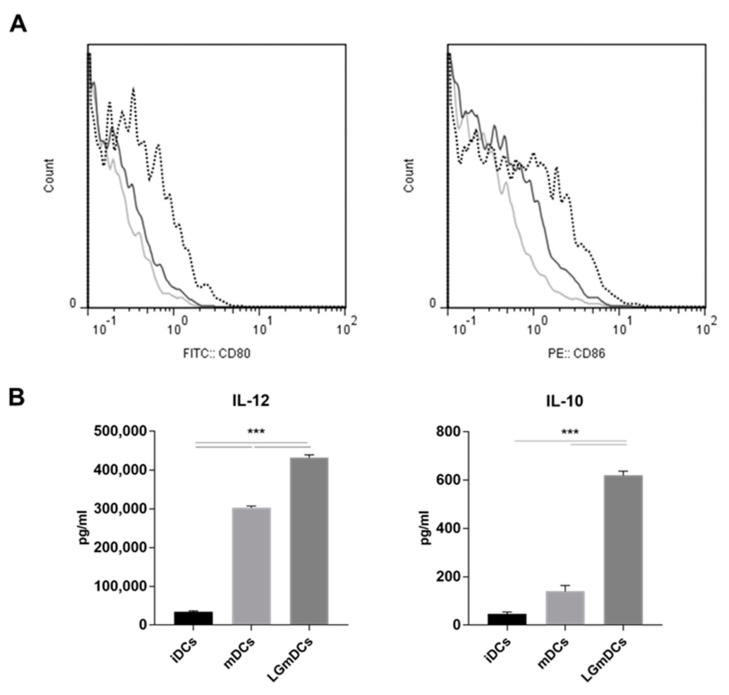
Phenotype of dendritic cells (DCs) cultured in X-Vivo 15 medium and challenged with *L. gasseri*. Immature dendritic cells (iDCs) were challenged or not with irradiated *L. gasseri* and then subjected to a 6-h lipopolysaccharide (LPS) pulse to induce DC maturation. (**A**) Fluorescence-activated cell sorting (FACS) analysis of DCs double-stained for CD80 and CD86; light grey line, iDCs; dark grey line, mDCs; dotted line, *L. gasseri* OLL2809 challenged iDCs (LGmDCs). (**B**) Cytokine analysis (Interleukin-10 (IL-10) and Interleukin-12 (IL-12))of iDCs, mature dendritic cells (mDCs), and LGmDCs. Data were collected from ungated cells and are representative of three independent experiments. *** *p* < 0.01 (ANOVA test).

A quantitative proteomic study was designed aimed at investigating both the maturation process in DCs and the effects of a *L. gasseri* challenge on this process. The study focused on secreted proteins, as these proteins (cytokines, chemokines, proteases, protease inhibitors, growth factors, etc.) play a major role in both innate and adaptive responses driven by DCs [15]. To obtain a more inclusive picture of the secretome, unspecific protein contribution from cell culture was drastically reduced by incubating iDCs in a serum-free medium. Specifically, in vitro generated iDCs were pre-incubated for 24 h in X-Vivo 15 medium [25].  In this medium, we found ≥ 90% live cells. Cell integrity was assessed by measuring the percentage LDH release in the spent medium and this assay showed that DC maturation was not associated with any decrease in cell integrity. In fact, iDCs and mDCs showed comparable values %LDH release (11.1 ± 0.8 vs. 10.6 ± 0.6; mDCs vs. iDCs; mean ± SD; P = 0.71) and LGmDCs showed a non-significant increase (14.6 + 2.7; P = 0.08). The increased expression of both CD80 and CD86 surface markers testified the effective maturation of DCs (Figure 1A). We also estimated the induced phenotype by evaluating the production of interleukin (IL)-12 and IL-10, a pro- and an anti-inflammatory cytokine, respectively. As reported in Figure 1B, IL-12 significantly increased in mDCs, whereas changes in IL-10 levels were not relevant. Therefore, induced mDCs had a pro-inflammatory signature. Interestingly, pre-incubation with *L. gasseri* further induced the expression of surface markers (Figure 1A) as well as increased the levels of IL-12 and IL-10 (Figure 1B); the latter findings confirmed the ability of this probiotic strain to modulate the phenotype of mDCs.

Moreover, the modulation of secretome profiles was investigated by proteomics analyzing the secretomes of iDCs, mDCs, and LGmDCs. This study led to the identification of 156 proteins included in the dataset that were reproducibly present in three biological replicates (out of four replicates analyzed in the study) of at least one sample (Table S1 (All Proteins) in Supplementary Materials).

Bioinformatics processing of the proteins included in the dataset using the SignalP tool highlighted that 33 proteins were predicted to contain a signal peptide for translocation across cell membranes. DeepLoc analysis predicted extracellular localization for 21 of these proteins, and for the other five proteins (Ctss, Il1rn, Ctsb, Pla2g7, Lgals1), the extracellular localization was deduced from Uniprot annotation. An additional 21 proteins were predicted to be non-classically secreted proteins by SecretomeP analysis. Therefore, 30% of the identified proteins were extracellularly located based on bioinformatics processing. However, it cannot be ruled out that other proteins identified in this study could be extracellularly located. For example, it has been reported that cathepsins, generally located in the lysosomes and endosomes, could be secreted into the extracellular milieu as proenzymes and have also been found in cell-cultured medium and in the circulation of both experimental animals and human beings under physiological conditions [26,27]. Similarly, peptidyl-prolyl cis-trans isomerase has been observed in the conditioned medium of lipopolysaccharide-stimulated murine macrophages [28,29]. In addition, due to partial cell lysis occurring during cell growth or sample preparation, minor cytoplasmic protein contamination should also be taken into consideration.

The functional classification of these proteins, performed using the Reactome Pathway Database, highlighted that the immune system, signal transduction, cellular responses to external stimuli, and vesicle-mediated transport were among the most enriched Reactome pathways. These data are in tight agreement with the physiology of DCs and their modification following stimuli (Table S3 in Supplementary Materials).

### 3.2. Secretome Analysis of the Immature DCs

A first overall picture of the secretome of iDCs was gained through proteomics leading to the identification of 61 proteins, and 21 of these (34%) were predicted to be extracellularly localized (Table S1 (iDCs) in Supplementary Materials). It is worth noting, all the proteins identified in the iDCs secretome were also present in the secretome of mDCs (Table S1 (mDCs) in Supplementary Materials). Similarly to data reported by Gundacker and colleagues [15], iDCs secreted several proteases and protease inhibitors (Cystatin-C (Cst3), Cathepsin B (Ctsb), Cathepsin D (Ctsd), Cathepsin L (Ctsl), Cathepsin S (Ctss), Cathepsin Z (Ctsz)) involved in antigen processing and Galectin 3 (Lgals3) involved in inflammatory responses including neutrophil activation and adhesion, as well as proteins involved in redox regulation (Prdx1 and Prdx5, Sod1) and in protein folding (Hsp90ab1, Hspa8, Ppia). In addition, actin, MMP12 (probably involved in cell trafficking), and peroxiredoxin 1 were already detected in the secretome of iDCs by Villiers and colleagues [16].

**Figure 2:**
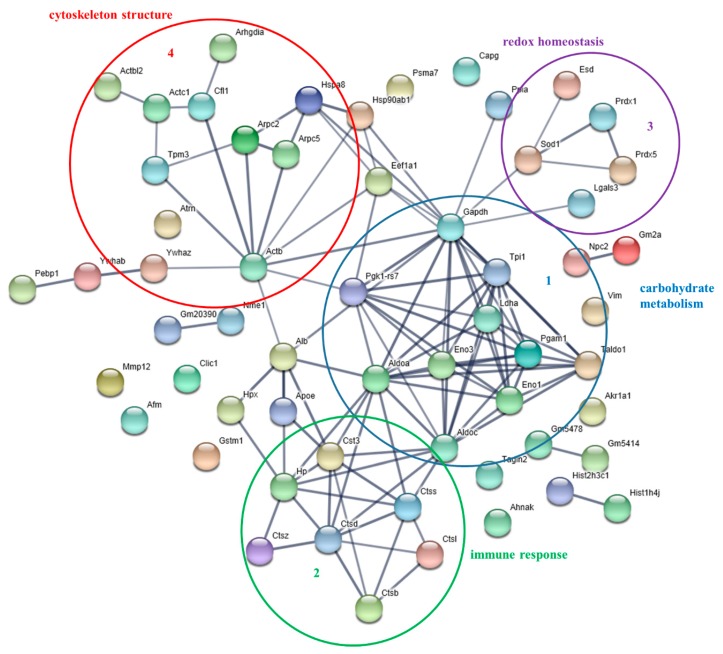
Protein–protein interaction network of proteins identified in the secretome of immature DCs. The Gene Name according to UniProtKB are reported. The network was obtained using the EMBL STRING with a confidence cut-off of 0.700 [24]. The four main functional modules are circled and numbered.

Protein–protein interactions were analyzed, processing data reported in Table S1 (iDCs), by means of the web resource STRING (Figure 2). Notably, STRING analysis highlighted highly interconnected clusters of proteins. The main clusters included proteins involved in carbohydrate catabolic processes (Cluster 1: Eno1, Eno2, Ldha, Aldoa, Aldoc, etc.), in immune response (Cluster 2: several cathepsins acting in protein degradation and turnover), in redox homeostasis (Cluster 3: Prdx1 and Prdx5, Sod1 and Esd) and finally, in cytoskeleton structure (Cluster 4: mainly joining actins and tropomyosin). More interestingly, coherent with the immunological role of iDCs, the Reactome Pathway analysis showed that the most enriched pathways of this dataset were “Innate Immune System” (MMU-168249, 23 proteins, fold enrichment + 8.37, FDR 6.6E-13), and in particular “Neutrophil degranulation” (including Cst3, Gm2a, Aldoa, Aldoc, Arpc5, Gm20390, Hp, Hsp90ab1, Npc2, Pgam1, Lgals3), and “Adaptive Innate Immune System” (MMU-1280218, 4 proteins, fold enrichment + 12.6, FDR 2.4E-2) and in particular “MHC class II antigen presentation”, including proteins of the cathepsin family (Ctss, Ctsd, Ctsb, Ctsl). (Table S3 (iDCs)).

### 3.3. Modulation of DCs Secretome Induced by the Maturation Process

**Figure 3:**
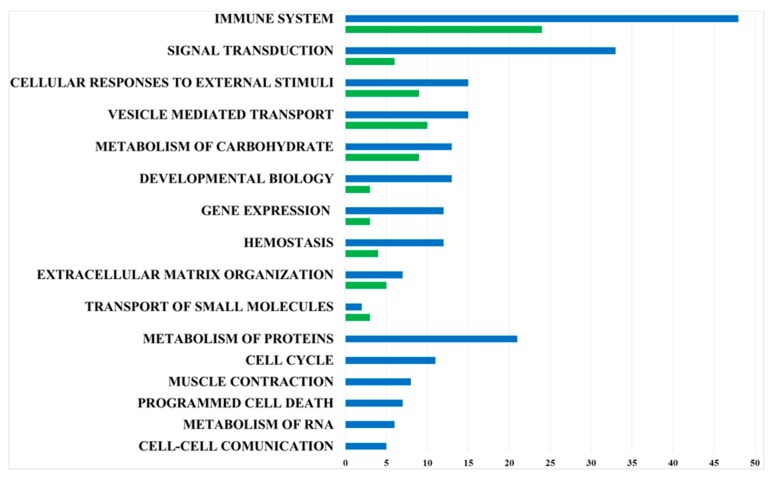
Bar diagram summarizing the functional classification of proteins present in the mDCs secretome (blue bars) and in the secretome of iDCs (green bars) achieved by Reactome Pathway Database analysis.

The proteomic analysis of mDCs secretome led to the identification of 130 proteins present in at least three biological replicates. Twenty proteins were predicted to be extracellularly located, and 18 proteins were secreted through non-classical pathways (29% extracellular proteins) (Table S1 (mDCs) in Supplementary Materials). The functional classification confirmed that the maturation process further enriched the secretome profile with proteins involved in immunity. In fact, 46 proteins were classified as belonging to “Innate Immune System” (MMU-168249, fold enrichment + 7.71, FDR 6.55E-25), including C3, Lcn2, Rock1, that plays a functional role in the dynamic reorganization of cytoskeletal proteins, while, for instance, only 23 proteins of the iDCs secretome were involved in “Innate Immune System”. Similarly, the maturation process led to the expression of a larger number of proteins involved in Signal Transduction (Figure 3, Table S3 (mDCs) in Supplementary Materials).

A quantitative proteomic analysis led to the identification of 37 proteins exhibiting a statistically significant variation in abundance when comparing the secretome of mDCs vs. iDCs. The maturation process triggered the abundance of 32 proteins and reduced the amount of five proteins in mDCs (Table 1; Table S2 in Supplementary Materials)).

**Figure 4:**
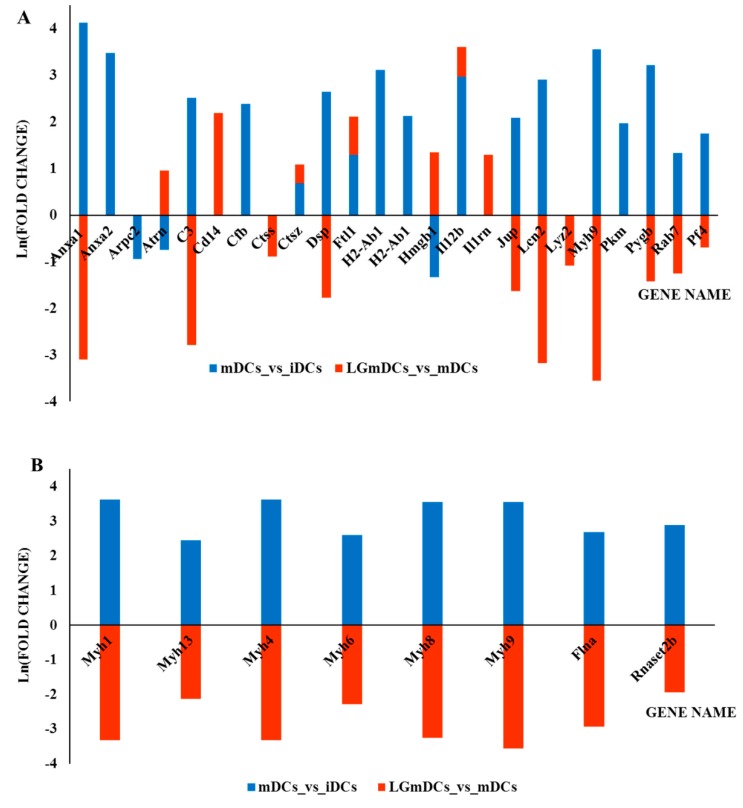
Bar diagram summarizing the most relevant results of the quantitative proteomic analyses and reporting the natural logarithm (Ln) of fold change values obtained comparing mDCs vs. iDCs and LGmDCs vs. mDCs. Fold change values are reported in Table 1. Positive values of Ln (fold change) indicate an increase in protein amount, while negative values of Ln (fold change) indicate a decrease in protein amount. (**A**) Proteins mainly involved in the immune response. (**B**) Proteins mainly involved in contractile machinery. These proteins showed a significant increase in fold changes when comparing mDCs vs. iDCs (blue bars) and a dramatic decrease when comparing LGmDCs vs. mDCs (red bars).

Functional classification of these proteins highlighted that proteins related to “Innate Immune System” (MMU-168249, 13 proteins, fold enrichment + 7.75 FDR 7.1E-06), such as Anxa2, Arpc2, C3, Ctsz, Dsp, Jup, Myh9, Pkm, Pygb, Rab7, were significantly more abundant in mDCs. In addition, the “Signaling by RHO GTPase” pathway was significantly enriched (MMU-194315, 5 proteins, fold enrichment + 12.24 FDR 1.5E-02) (Figure 4A, Table 1, Table S3 (mDCs vs. iDCs) in Supplementary Materials).

Proteins involved in intracellular transport, cell–cell communication, and cytoskeletal proteins, in particular myosin isoforms, tropomyosin and filamin, were highly abundant in mDCs compared to iDCs, thus indicating that DCs are preparing for migration, which requires profound alterations in cell morphology and motility (Figure 4B).

IL-12 subunit beta was present in the mDCs secretome and absent in the iDCs one, while IL-10 was not detected in this analysis, probably due to the instrumental detection limit. These results are in tight agreement with the ELISA data (Figure 1B). Interestingly, we detected an increased abundance of canonical extracellular proteins with general anti-microbial activity, such as Complement 3 (C3), Complement factor b (Cfb), and Lipocalin-2 (Chromosome 24p3, Lcn2). We also detected an increased presence of proteins with more specific roles, such as Platelet aggregating factor 4 (Pf4) and Serum amyloid A3 protein (Saa3). The latter one stimulates neutrophils to secrete IL-22 for protecting the integrity of the epithelial barrier [30]. The pleiotropic role of mDCs in the immunological synapse was confirmed by the massive presence of sMHC II (H2-Ab1), Annexins, and extracellular released Histone 2, known to engage the T cell receptor or toll-like receptors. Through these molecules, both the innate and adaptive arms of the immune response could be activated (Table 1). Furthermore, the increased abundance of contractile machinery proteins (myosin polypeptide, filamin-A, tropomyiosin, Tpm2) and desmosome proteins (desmoplakin, junction plakoglobin) could testify an increased migration/cell interaction activity of mDCs. Unexpectedly, we also registered an increased abundance of eukaryotic initiation factor 5A (eIF-5A). In DCs, eIF-5A is mainly involved in the intracellular transport pathway of transcript encoding for CD83, a maturation marker [31]. Noteworthy, in cardiac myocytes, eIF5A undergoes tyrosine sulfation in the trans-Golgi and is rapidly secreted in response to hypoxia, inducing apoptosis via an autocrine mechanism [32]. Therefore, our findings suggested that secreted eIF5A could work as a pro-apoptotic ligand in different cell types. Intriguingly, the analysis also revealed the presence of enzymes generally found intracellularly in the secretome of mDCs (Pkm, Ppib, Pyg). Several non-canonical functions have already been attributed to such enzymes. In particular, neutrophils at tissue damaged sites could release pyruvate kinase m1/2 to promote angiogenesis and wound healing [33]. Furthermore, extracellular cyclophilins (peptidylprolyl isomerase) have been shown to modulate the levels of TNFα [29]. The increased presence of RNase T2 could also be expected, as it degrades RNAs in the extracellular space, thus playing a role in innate host defense. On the contrary, the secretion of glycogen phosphorylases (Pyg), classical intracellular enzymes, remains to be clarified.

### 3.4. Modulation of the Secretome of mDCs by L. gasseri Challenge

To further elucidate the modulatory effects induced by this probiotic strain on the maturation process, the secretomes of LGmDCs and mDCs were compared. The microbial treatment affected the abundance level of 37 proteins, increasing the level of 13 proteins and decreasing that of 24 proteins. These proteins were functionally classified in “Immune System” (fold enrichment + 4.77, FDR 2.3E-04, 14 proteins), in “Platelet activation, signaling, and aggregation” (MMU-76002, fold enrichment + 17.22, FDR 3.0E-02, four proteins: Pf4, Flna, Fn1, Tagln2) and “Extracellular matrix organization” (MMU-1474244, fold enrichment + 9.50, FDR 4.2E-02, five proteins: Ctss, Ppib, Mmp12, Fn1, Dsp) (Figure 4, Table S3 (LGmDCs vs. mDCs) in Supplementary Materials).

Most importantly, *L. gasseri* pre-treatment was associated with a dramatic decrease of 19 proteins that mainly featured the mDC secretome, thanks to their relatively high abundance (as from LGmDCs vs. mDCs and mDCs vs. iDCs comparisons). In particular, we found that not only proteins closely related to the immune function (Annexins, C3, Lcn2) but also contractile machinery proteins (Myosins, Filamin-A) and desmosome proteins were drastically reduced (Figure 4). The abundance of Il12b and Ctsz was instead increased in both the comparisons (Table 1, Figure 4).

In addition, seven proteins (Tagln2, Ppib, Coro1a, Il1rn, Esd, Hnrnpa, CD14) were specifically involved in the maturation process mediated by *L. gasseri* (Table1), increasing their amount. In particular, the increased abundance of Interleukin-1 receptor antagonist protein, an early inhibitory cytokine that suppresses pro-inflammatory cytokines and Monocyte differentiation antigen CD14, a co-receptor for the detection of LPS and other pathogen-associated molecular patterns (i.e., lipoteichoic acid), could suggest a possible protective role of LG treatment against inflammation. Recently, Transgelin-2 (Tagln2) has been recognized as an important immune regulator in the context of immune cell-cell interaction [34] and is suggested to be also involved in the stability of the immunological synapse in B-cells [35]. Coronin (Coro1a, a component of the cytoskeleton of highly motile cells) plays a key role in modulating the activity of lymphocytes, neutrophils, and macrophages [36]. Finally, the preventive bacterial challenge caused a strong reduction of fibronectin, classically involved in different cell adhesion and migration processes (Table 1).

## 4. Conclusions

The present proteomic study established that the maturation process of DCs was associated with profound changes in the protein secretome. These changes went beyond the increased expression of classical immune mediators (i.e., cytokines, complement factors, TcR ligands), but involved some unexpected new mediators (i.e., eIF5A), as well as the contractile and desmosome machineries. The latter findings will help to explain the role of DCs in the current cell migration model, where integrin adhesions are built upon initial cell contact with the matrix, followed by the generation of traction forces on the adhesions through local actomyosin-based contractile units. *L. gasseri* pre-treatment was associated with a dramatic modulation of several proteins secreted by mDC, not only belonging to the immune synapse. In fact, *L. gasseri* appeared to reduce junction formation and the secretion of actomyosin-based contractile units. It is known that *L. gasseri* drives a physiological activity for regulating cell adherence and integrating/activating or controlling signals in dendritic and T/B cell networks. Therefore, these findings suggest a novel mechanism by which *L. gasseri* could modulate the maturation process of DCs, thus confirming that this probiotic strain exerts a protective anti-inflammatory role. Further studies are needed to investigate whether these findings reflect the general properties of probiotic strains.

## Figures and Tables

**Table 1:** Results of the differential proteomic study: proteins whose abundance was affected by the maturation process of DCs challenged or not with *L. gasseri* and functional classification obtained by the Reactome Pathway Database.

ACCESSION UNIPROTKB	DESCRIPTION	GENE NAME	FUNCTIONAL CLASSIFICATION	FOLD CHANGE	
				mDCs/iDCs	LGmDCs/mDCs
			**IMMUNE SYSTEM**		
Q542G9	Annexin 2	Anxa2	Innate Immune System Neutrophil degranulation	32.54	
P01027	Complement C3	C3	Innate Immune System Neutrophil degranulation	12.29	−16.16
Q3U6A3	Monocyte differentiation antigen CD14	Cd14	Innate Immune System Neutrophil degranulation		8.91
Q3UD32	Uncharacterized protein	Ctss	Innate Immune System Neutrophil degranulation		−2.42
Q9ES94	Cathepsin Z	Ctsz	Innate Immune System Neutrophil degranulation	1.97	1.53^a^
E9Q557	Desmoplakin	Dsp	Innate Immune System Neutrophil degranulation	14.13	−5.86
Q3UBK2	Uncharacterized protein	Hmgb1	Innate Immune System Neutrophil degranulation	−3.80	3.85
Q02257	Junction plakoglobin	Jup	Innate Immune System Neutrophil degranulation	8.07	−5.13
P08905	Lysozyme C-2	Lyz2	Innate Immune System Neutrophil degranulation		−2.97
Q3TFQ8	Alpha-1,4 glucan phosphorylase	Pygb	Innate Immune System Neutrophil degranulation	25.10	−4.13
Q4FJQ0	MCG130610	Rab7	Innate Immune System Neutrophil degranulation	3.80	−3.52
Q60842	Chromosome 24p3 (Fragment)	Lcn2	Innate Immune System Neutrophil degranulation	18.34	−24.10
Q5FWA0	Ribonuclease T2B	Rnaset2b	Innate Immune System Neutrophil degranulation	17.80	−6.96
B8JJN0	Uncharacterized protein	Cfb	Innate Immune System	10.86	
P43432	Interleukin-12 subunit beta	Il12b	Cytokine Signaling in Immune System	19.36	1.89^a^
P25085	Interleukin-1 receptor antagonist protein	Il1rn	Cytokine Signaling in Immune System		3.65
P29391	Ferritin light chain 1	Ftl1	**IMMUNE SYSTEM**	3.63	−2.28
P01921	H-2 class II histocompatibility antigen, A-D beta chain	H2-Ab1	**IMMUNE SYSTEM**	22.45	
P06343	H-2 class II histocompatibility antigen, A-K beta chain	H2-Ab1	**IMMUNE SYSTEM**	8.33	
					
			**SIGNAL TRANSDUCTION**		
Q64426	Histone H2A (Fragment)	Hist2h2aa1	Signaling by Nuclear Receptors	12.83	
Q6P8R3	C-X-C motif chemokine	Pf4	Signaling by GPCR	5.72	−1.98^a^
Q3US43	Annexin 1	Anxa1	Signaling by GPCR	61.76	−22.14
P60766	Cell division control protein 42 homolog	Cdc42	Signaling by Rho GTPases		-2.88
Q3THE2	Myosin regulatory light chain 12B	Myl12b	Signaling by Rho GTPases /**MUSCLE CONTRACTION** Smooth Muscle Contraction	3.80	
					
			**MUSCLE CONTRACTION**		
Q91VH3	Tpm2 protein	Tmp2	Striated Muscle Contraction Smooth Muscle Contraction	4.71	−2.20
F8WID5	Tropomyosin alpha-1 chain	Tpm1	Striated Muscle Contraction Smooth Muscle Contraction	3.55	
B2RQQ1	MCG133649, isoform CRA_a	Myh6	Striated Muscle Contraction	13.33	−9.83
B2RWW8	Myosin, heavy polypeptide 8	Myh8	Striated Muscle Contraction	35.12	−25.91
B2RWX0	Myosin, heavy polypeptide 1	Myh1	Striated Muscle Contraction	37.49	−27.66
B1AR69	Myosin, heavy polypeptide 13	Myh13	Striated Muscle Contraction	11.44	−8.44
Q5SX39	Myosin-4	Myh4	Striated Muscle Contraction	37.62	−27.75
					
			**HEMOSTASIS**		
Q8BTM8	Filamin-A	Flna	Platelet activation, signaling, and aggregation	14.59	−18.85
P11276	Fibronectin	Fn1	Platelet activation, signaling, and aggregation		−74.75
Q9WVA4	Transgelin-2	Tagln2	Platelet activation, signaling, and aggregation		2.30
					
			**EXTRACELLULAR MATRIX ORGANIZATION**		
P34960	Macrophage metalloelastase	Mmp12	Degradation of extracellular matrix	−1.80^a^	4.47
P24369	Peptidyl-prolyl cis-trans isomerase B	Ppib	Collagen Formation		3.44
					
			**METABOLISM**		
H3BKH6	S-formylglutathione hydrolase	Esd	Biological Oxidations		2.08
P08226	Apolipoprotein E	Apoe	Metabolism of vitamins and cofactors		−3.64
Q9WUB3	Glycogen phosphorylase	Pygm	Metabolism of carbohydrate	51.35	−10.93
P63242	Eukaryotic translation initiation factor 5A-1	Eif5a	Metabolism of proteins Post-translational protein modification	13.51	
A2AB79	Histone H2A	Hist3h2a	Metabolism of proteins Post-translational protein modification	11.72	
					
			**UNCLASSIFIED**		
Q3U1N0	Coronin	Coro1a			2.52
Q20BD0	Heterogeneous nuclear ribonucleoprotein A/B, isoform CRA_a	Hnrnpa			7.24
P04918	Serum amyloid A-3 protein	Saa3		14.27	
Q99LB4	Capping protein (Actin filament), gelsolin-like, isoform CRA_a	Capg		−2.33	1.84^a^
B9EIU3	Attractin	Atrn		−2.10	2.61

^a^ proteins with fold change values in the range ± 1.5–2.0.

## Supplementary Materials

The following are available online at https://www.mdpi.com/2072-6643/12/2/555/s1, Table S1: Proteomic data gathered in this study. List of the proteins identified in this study. The accession number (UniProtKB), the gene name and cell localization are reported together with the number of matched peptides and peptide mass spectra matches (PSM) for each protein in each analyzed sample (All proteins sheet); List of the proteins identified in the secretome of iDCs (iDCs sheet); List of the proteins identified in the secretome of mDCs (mDCs sheet); Quantitative label-free analysis performed on the proteomic data applying the spectral counting approach (Quantitation_analysis sheet); Gene names for some of the proteins included in functional analyses as deduced using BLASTP (https://blast.ncbi.nlm.nih.gov) (Blast results sheet); Table S2: Results of the differential proteomic study: proteins whose abundance was affected by the maturation process of DCs challenged or not with *L. gasseri*; Table S3: Functional analysis of the proteins identified in the proteomic study. Reactome Pathway analysis of proteins identified in the secretome of iDCs (iDCs sheet); Reactome Pathway analysis of proteins identified in the secretome of mDCs (mDCs sheet); Reactome Pathway analysis of proteins involved in the maturation process (mDCs vs. iDCs sheet); Reactome Pathway analysis of proteins involved in the maturation process modulated by L. gasseri (LGmDCs vs. mDCs sheet).
