# Supplementary material for: Secretome Analysis of Mouse Dendritic Cells Interacting with a Probiotic Strain of Lactobacillus gasseri
Source: Nutrients. 2020 Feb 20;12(2):555. doi: 10.3390/nu12020555 (PMC7071482; doi:10.3390/nu12020555)
Supplement: Supplementary file 1 [file nutrients-12-00555-s001.zip › Table S2.docx]

**Table S2:** Results of the differential proteomic study: proteins whose abundance was affected by the maturation process of DCs challenged or not with *L.* *gasseri.*

| **Accession**  **UniProtKB** | **Description** | **Gene name** | **mDCs/iDCs** | | **LGmDCs/mDCs** | | **Cell localization**  **(DeepLoc/**  **UniProt)** | **Secretome P PREDICTION** |
| --- | --- | --- | --- | --- | --- | --- | --- | --- |
|  |  |  | **Fold Change ^a^** | **P value** | **Fold Change ^a^** | **P value** |  |  |
| Q3US43 | Annexin 1 | Anxa1 | 61.76 | 0.012 | -22.14 | 0.040 |  |  |
| Q542G9 | Annexin 2 | Anxa2 | 32.54 | 0.014 |  |  |  | Non- classically secreted proteins |
| P08226 | Apolipoprotein E | Apoe |  |  | -3.64 | 0.0001 | Extracellular |  |
| Q3UA52 | Arp2/3 complex 34 kDa subunit (Fragment) | Arpc2 | -2.57 | 0.020 |  |  |  | Non- classically secreted proteins |
| B9EIU3 | Attractin | Atrn | -2.10 | 0.080 ^c^ | 2.61 | 0.037 |  |  |
| P01027 | Complement C3 | C3 | 12.29 | 5.6E-05 | -16.16 | 2.5E-05 | Extracellular |  |
| Q99LB4 | Capping protein (Actin filament), gelsolin-like, isoform CRA_a | Capg | -2.33 | 0.013 | 1.84 ^b^ | 0.092 ^c^ |  |  |
| Q3U6A3 | Monocyte differentiation antigen CD14 | Cd14 |  |  | 8.91 | 0.007 | Extracellular |  |
| P60766 | Cell division control protein 42 homolog | Cdc42 |  |  | -2.88 | 0.048 |  | Non- classically secreted proteins |
| B8JJN0 | Uncharacterized protein | Cfb | 10.86 | 0.007 |  |  | Extracellular |  |
| Q3U1N0 | Coronin | Coro1a |  |  | 2.52 | 0.030 |  | Non- classically secreted proteins |
| Q3UD32 | Uncharacterized protein | Ctss |  |  | -2.42 | 0.019 | Extracellular |  |
| Q9ES94 | Cathepsin Z | Ctsz | 1.97 | 0.011 | 1.53 ^b^ | 0.073 ^c^ | Extracellular |  |
| E9Q557 | Desmoplakin | Dsp | 14.13 | 0.027 | -5.86 | 0.100 ^c^ |  |  |
| P63242 | Eukaryotic translation initiation factor 5A-1 | Eif5a | 13.51 | 2.7E-05 |  |  |  |  |
| H3BKH6 | S-formylglutathione hydrolase | Esd |  |  | 2.08 | 0.009 |  | Non- classically secreted proteins |
| Q8BTM8 | Filamin-A | Flna | 14.59 | 0.0050 | -18.85 | 0.003 |  |  |
| P11276 | Fibronectin | Fn1 |  |  | -74.75 | 0.002 | Extracellular |  |
| P29391 | Ferritin light chain 1 | Ftl1 | 3.63 | 0.031 | -2.28 | 0.100 ^c^ |  |  |
| P01921 | H-2 class II histocompatibility antigen, A-D beta chain | H2-Ab1 | 22.45 | 0.0007 |  |  |  |  |
| P06343 | H-2 class II histocompatibility antigen, A-K beta chain | H2-Ab1 | 8.33 | 0.0006 |  |  |  |  |
| Q64426 | Histone H2A (Fragment) | Hist2h2aa1 | 12.83 | 0.045 |  |  |  |  |
| A2AB79 | Histone H2A | Hist3h2a | 11.72 | 0.045 |  |  |  |  |
| Q3UBK2 | Uncharacterized protein | Hmgb1 | -3.80 | 0.046 | 3.85 | 0.044 |  |  |
| Q20BD0 | Heterogeneous nuclear ribonucleoprotein A/B, isoform CRA_a | Hnrnpa |  |  | 7.24 | 0.0024 |  |  |
| P43432 | Interleukin-12 subunit beta | Il12b | 19.36 | 1.2E-05 | 1.89 ^b^ | 0.032 | Extracellular |  |
| P25085 | Interleukin-1 receptor antagonist protein | Il1rn |  |  | 3.65 | 0.0005 | Extracellular |  |
| Q02257 | Junction plakoglobin | Jup | 8.07 | 0.037 | -5.13 | 0.069 ^c^ |  |  |
| Q60842 | Chromosome 24p3 (Fragment) | Lcn2 | 18.34 | 3.7E-05 | -24.10 | 1.8E-05 | Extracellular |  |
| P08905 | Lysozyme C-2 | Lyz2 |  |  | -2.97 | 0.0018 | Extracellular |  |
| P34960 | Macrophage metalloelastase | Mmp12 | -1.80 ^b^ | 0.100 ^c^ | 4.47 | 0.0005 | Extracellular |  |
| B2RWX0 | Myosin, heavy polypeptide 1, | Myh1 | 37.49 | 0.004 | -27.66 | 0.010 |  |  |
| B1AR69 | Myosin, heavy polypeptide 13, | Myh13 | 11.44 | 0.032 | -8.44 | 0.071 ^c^ |  |  |
| Q5SX39 | Myosin-4 | Myh4 | 37.62 | 0.004 | -27.75 | 0.009 |  |  |
| B2RQQ1 | MCG133649, isoform CRA_a | Myh6 | 13.33 | 0.013 | -9.83 | 0.032 |  |  |
| B2RWW8 | Myosin, heavy polypeptide 8, | Myh8 | 35.12 | 0.003 | -25.91 | 0.007 |  |  |
| Q8VDD5 | Myosin-9 | Myh9 | 35.16 | 0.015 | -35.01 | 0.015 |  |  |
| Q3THE2 | Myosin regulatory light chain 12B | Myl12b | 3.80 | 0.018 |  |  |  |  |
| Q6P8R3 | C-X-C motif chemokine | Pf4 | 5.72 | 0.013 | -1.98 | 0.100 ^c^ | Extracellular |  |
| P52480 | Pyruvate kinase | Pkm | 7.19 | 0.040 |  |  |  |  |
| P24369 | Peptidyl-prolyl cis-trans isomerase B | Ppib |  |  | 3.44 | 0.001 |  |  |
| Q3TFQ8 | Alpha-1,4 glucan phosphorylase | Pygb | 25.10 | 1.2E-05 | -4.13 | 0.033 |  |  |
| Q9WUB3 | Glycogen phosphorylase | Pygm | 51.35 | 9.7E-06 | -10.93 | 0.008 |  |  |
| Q4FJQ0 | MCG130610 | Rab7 | 3.80 | 0.038 | -3.52 | 0.069 ^c^ |  |  |
| Q5FWA0 | Ribonuclease T2B | Rnaset2b | 17.80 | 6.9E-06 | -6.96 | 0.005 | Extracellular |  |
| P04918 | Serum amyloid A-3 protein | Saa3 | 14.27 | 0.002 |  |  | Extracellular |  |
| Q9WVA4 | Transgelin-2 | Tagln2 |  |  | 2.30 | 0.021 |  | Non- classically secreted proteins |
| Q91VH3 | Tpm2 protein | Tmp2 | 4.71 | 0.013 | -2.20 | 0.050 |  |  |
| F8WID5 | Tropomyosin alpha-1 chain | Tpm1 | 3.55 | 0.028 |  |  |  | Non- classically secreted proteins |
